# Assessing Structural Racism and Discrimination Along the Pre-exposure Prophylaxis Continuum: A Systematic Review

**DOI:** 10.1007/s10461-024-04387-y

**Published:** 2024-06-08

**Authors:** Sara Seyedroudbari, Fatemeh Ghadimi, Gabriela Grady, Obinna Uzosike, Hervette Nkwihoreze, John B. Jemmott, Florence Momplaisir

**Affiliations:** 1 College of Medicine, Drexel University, Philadelphia, PA USA; 2 Division of Infectious Diseases, Department of Medicine, Perelman School of Medicine, University of Pennsylvania, Philadelphia, PA USA; 3 Bryn Mawr College, Bryn Mawr, PA USA; 4 Princeton University, Princeton, NJ USA; 5 Annenberg School for Communication, University of Pennsylvania, Philadelphia, PA USA; 6 Department of Psychiatry, Perelman School of Medicine, University of Pennsylvania, Philadelphia, PA USA; 7 Leonard Davis Institute of Health Economics, University of Pennsylvania, Philadelphia, PA USA

**Keywords:** HIV, Pre-exposure prophylaxis (PrEP), Racism, Race and structural racism, Systemic discrimination

## Abstract

Structural racism and discrimination (SRD) is deeply embedded across U.S. healthcare institutions, but its impact on health outcomes is challenging to assess. The purpose of this systematic literature review is to understand the impact of SRD on pre-exposure prophylaxis (PrEP) care continuum outcomes across U.S. populations who could benefit from HIV prevention. Guided by PRISMA guidelines, we conducted a systematic review of the published literature up to September 2023 using PubMed and PsycInfo and included peer-reviewed articles meeting inclusion criteria. At least two authors independently screened studies, performed quality assessments, and abstracted data relevant to the topic. Exposure variables included race/ethnicity and any level of SRD (interpersonal, intra- and extra-organizational SRD). Outcomes consisted of any steps of the PrEP care continuum. A total of 66 studies met inclusion criteria and demonstrated the negative impact of SRD on the PrEP care continuum. At the interpersonal level, medical mistrust (i.e., lack of trust in medical organizations and professionals rooted from current or historical practices of discrimination) was negatively associated with almost all the steps across the PrEP care continuum: individuals with medical mistrust were less likely to have PrEP knowledge, adhere to PrEP care, and be retained in care. At the intra-organizational level, PrEP prescription was lower for Black patients due to healthcare provider perception of higher sex-risk behaviors. At the extra-organizational level, factors such as homelessness, low socioeconomic status, and incarceration were associated with decreased PrEP uptake. On the other hand, healthcare provider trust, higher patient education, and access to health insurance were associated with increased PrEP use and retention in care. In addition, analyses using race/ethnicity as an exposure did not consistently show associations with PrEP continuum outcomes. We found that SRD has a negative impact at all steps of the PrEP care continuum. Our results suggest that when assessing the effects of race/ethnicity without the context of SRD, certain relationships and associations are missed. Addressing multi-level barriers related to SRD are needed to reduce HIV transmission and promote health equity.

**Supplementary Information:**

The online version contains supplementary material available at 10.1007/s10461-024-04387-y.

## Introduction

Structural racism is deeply embedded in the structures and institutions of U.S. society. Structural racism and discrimination (SRD) refers to the conditions that limit opportunities, resources, power, and well-being of individuals and populations based on race and ethnicity and other statuses leading to poorer health outcomes [1]. Health disparities have been demonstrated across many racial and ethnic groups, notably Black, Latinx, American Indian, and Alaska Native people, who experience higher rates of cardiovascular disease, diabetes, asthma, hypertension, and obesity than their White counterparts, in large part due to structures and systems of resource allocation (e.g. poor access to healthcare, education, and healthy food) stemming from SRD [2]. In addition to these chronic health conditions, HIV incidence is disproportionately high among Black and Latinx individuals, especially men who have sex with men (MSM), even almost 30 years after the pandemic’s peak [3]. This disparity in HIV incidence is further complicated by the potential for intersectional discrimination based on gender and sexuality.

Approved by the Food and Drug Administration (FDA) in 2012, oral Pre-exposure Prophylaxis (PrEP), when taken as prescribed, provides greater than 90 percent protection against acquiring HIV [4]. The newer developments of injectable PrEP, given every two months as opposed to a daily regimen [5, 6], and the PrEP “On-Demand” dosing regimen, taken 2–24 h before and 24–48 h after a potential HIV exposure[7] have expanded the accessibility of PrEP. Despite its high efficacy, multiple methods of administration, and a variety of dosing schedules, PrEP use is lowest among Black and Latinx people, although these groups have the highest HIV incidence rates. In 2022, only 8% of Black people eligible for PrEP were prescribed it, compared to 14% of Latinx people, and 60% of White people [8]. The steps of the PrEP continuum include increasing PrEP awareness, PrEP prescription, initiation and use of PrEP, PrEP adherence, and retention in care [9]. Each step can become a barrier to access for marginalized groups, leading to the low rates of PrEP usage in comparison to White individuals.

One limitation of current approaches to reducing disparities in PrEP usage is that most studies treat race/ethnicity as a risk factor for poor health outcomes but do not consider lived experiences with SRD. Using SRD along with race/ethnicity as an exposure leads to a more accurate conversation surrounding the impact of structural factors on disparities in the PrEP care continuum. Despite the variety of successful interventions that aim to reduce this well-documented disparity in PrEP usage, the persistence of the disparity asks us to reexamine our understanding and frameworks of how and why it continues to exist.

We aim to review the current literature concerning the impact of SRD on access to PrEP care for Black and Latinx people, with the goal of addressing existing gaps and retaining patients in the PrEP care continuum. The goal of this systematic review of the published literature is to assess peer-reviewed studies that have used SRD and/or race/ethnicity as the exposure in PrEP-related health outcomes to better understand the enduring disparities in the PrEP continuum and inform interventions that can be tailored to address these disparities.

## Methods

We followed the guidelines set forth by Preferred Reporting Items for Systematic Reviews and Meta-Analyses (PRISMA) for systematic reviews. Additionally, we registered this systematic review with PROSPERO (registration number: CRD42022350803), an international database of prospective systematic reviews. After a preliminary review of the published literature, we consolidated a list of relevant keywords and used these to create our unique search string based on the PICO (patient, population, or problem; intervention; comparison; outcome) framework for systematic reviews.

### Defining Exposures and Outcomes

Our exposures included self-reported race/ethnicity and multi-level exposures to SRD. SRD operates across distinct yet interconnected levels, and we have chosen to examine the interpersonal (first level), intra-organizational (second level), and extra-organizational (third level) [10]. The interpersonal level involves personal beliefs or stereotypes that influence interactions between individuals, such as the use of insensitive language or differential treatment recommendations. The intra-organizational level encompasses discriminatory practices within institutions, including clinic policies and organizational procedures that perpetuate racial inequities. SRD at the extra-organizational level deals with the cumulative effects of historical, cultural, and policy-driven mechanisms that perpetuate racial disparities through factors like access to education, economic opportunities, immigration policies, housing instability, and neighborhood conditions.

**Fig. 1:**
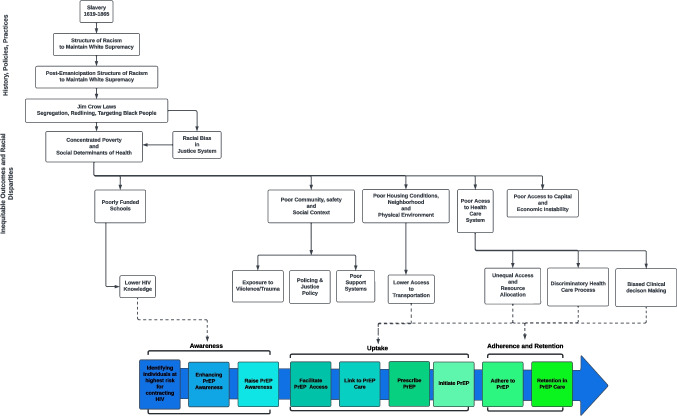
PrEP Care Continuum in the context of SRD

Regarding outcomes, our study assessed quantifiable effects on each stage of the PrEP care continuum (Fig. 1). The PrEP care continuum encompasses the full spectrum of care and support provided to individuals who are using PrEP and includes: PrEP awareness, uptake, adherence, and retention. We also included studies that evaluated HIV testing, such as previous testing and future intentions to get tested. As a negative test is needed to confirm serostatus before beginning PrEP, this outcome is pertinent to the PrEP care continuum. Lastly, the PrEP-to-Need Ratio, which assesses the adequacy of PrEP utilization, was included as an outcome relevant to the PrEP care continuum [11].

#### Search Strategy

A comprehensive search strategy was devised to identify relevant studies. The search was conducted using electronic databases, including PubMed and PsycINFO, to ensure coverage of medical, psychological, and social science literature. The search strategy was updated in September 2023 to include the latest research findings and ensure the most up-to-date information. The key search terms and keywords included but were not limited to: "HIV Pre-exposure Prophylaxis", "Racism", "Discrimination", "Health Disparities". The search strategy was developed using a combination of Medical Subject Headings (MeSH) terms, Boolean operators, and free-text terms to capture all relevant articles (full search strategy found in supplemental materials). The search was restricted to articles published in English.

#### Study Selection

The initial search yielded a comprehensive list of articles related to the impact of SRD on the PrEP care continuum. Two reviewers independently screened the titles and abstracts to identify relevant articles. For abstracts where there was not a consensus on relevance, a third reviewer was invoked to review the full-text article and resolve the conflict. Our team then evaluated the full-text articles of the selected abstracts to assess their eligibility for inclusion in the systematic review. We used the following selection criteria:

1. Studies that examined the impact of SRD on any of the steps of the PrEP care continuum.
2. We included quantitative and mixed-method studies that measured the impact of SRD across the PrEP care continuum. We excluded purely qualitative studies to focus on quantitative methods which are able to capture population-level data.
3. Studies that were based in the U.S. and published in peer-reviewed journals from January 2012 through September 2023, as the use of fixed dose combination of emtricitabine/tenofovir disoproxil fumarate as PrEP was approved by the U.S. Food and Drug Administration in 2012.

#### Data Extraction and Synthesis

Two reviewers extracted relevant data independently using a standardized data extraction form. The following information was captured from each included study and entered into a table in Microsoft Excel: study characteristics (author, year of publication, study design), participant characteristics (sample size, demographic information), measures or instruments used to assess SRD, PrEP care continuum outcomes, and findings related to the impact of SRD on PrEP outcomes.

#### Quality Assessment

Four reviewers independently evaluated the risk of bias and the overall quality of each study using the National Institutes of Health (NIH) Quality Assessment Tool for Observational Cohort and Cross-Sectional Studies [12] and the Newcastle–Ottawa Scale (NOS) for nonrandomized studies such as case control and cohort studies [13]. Using both scales, we assessed quality of the study question, sampling method, measurement of exposures, control for confounding variables, and adequacy of follow-up if applicable. Any discrepancies in quality assessment were resolved through discussion and consensus.

#### Data Analysis

We summarized and presented the results thematically across the steps of the PrEP care continuum.

## Results

**Fig. 2:**
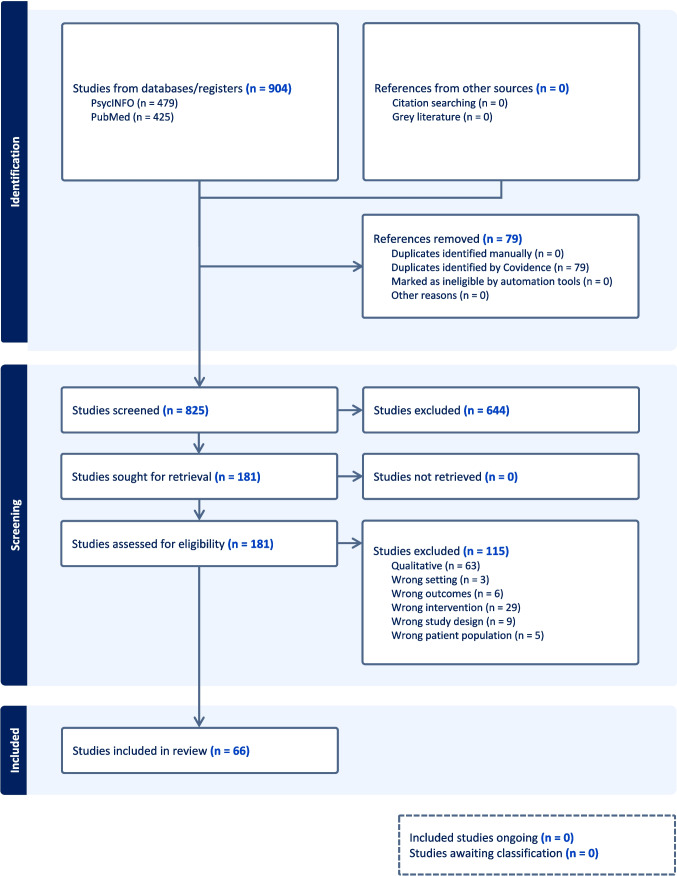
PRISMA flow diagram: systematic review of SRD and the PrEP care continuum

Out of 904 studies, 66 met the inclusion criteria and were included in the review (Fig. 2). The initial PubMed search retrieved 479 articles, and PsycInfo retrieved 425 articles. Upon reviewing abstracts and after duplicates were removed, we included 181 articles to review for further assessment of inclusion criteria. Upon reviewing the full texts, an additional 115 articles were excluded, and the remaining 66 peer-reviewed studies were included in our analysis.

### Types of Participants and Settings

Table 1 provides a comprehensive overview of included studies. The composition of participants in the included studies varied, with the majority consisting of PrEP users and/or individuals eligible for PrEP. Additionally, a handful of studies evaluated healthcare providers [14, 15] and medical or pharmacy students [16–20] as primary subjects. Participant demographics encompassed diverse intersections of racial/ethnic groups, gender identities, and sexual orientations, most prominently Latinx, Black, MSM, sexual minority men (SMM), transgender women (TW). White participants were frequently employed as the primary comparator group for statistical analysis, allowing for comparisons and assessments of racial/ethnic disparities in PrEP care.

**Table 1:** Causes of oesophageal perforation

First author, year	Purpose of the study	Study design	Location	Sampling strategy	Sample description, N	SRD measure (s)	PrEP outcome(s) and measure(s)	Effect measures and findings
Bauermeister, 2013 [47]	To gauge young MSM’s PrEP awareness and PrEP-related beliefs regarding side effects, accessibility, and affordability	Cross-sectional survey	United States (including Puerto Rico)	Participants recruited through advertisements on two popular social networking sites and participant referrals	1507 young MSM	Sociodemographic information including race/ethnicity, insurance, housing stability, and incarceration history (extra-organizational level SRD)	PrEP knowledge, perceived barriers to PrEP use	Young MSM were more likely to have heard about PrEP if they were older, more educated, were residentially unstable in the prior 30 days, had insurance, or reported having at least one STI in their lifetime. There was no association between PrEP awareness and race/ethnicity, recent sexual risk behavior, or incarceration. Young BLMSM were more likely than young White MSM to avoid PrEP due to side effect concerns. Young MSM who had insurance or did not report a prior STI were less likely to believe that they couldn’t afford PrEP
Bogart, 2019 [41]	To examine the prevalence and ecological, sociodemographic, and behavioral correlates of holding HIV conspiracy beliefs among Black Americans	Cross-sectional survey	United States	Participants recruited using a probability-based Web panel (GfK’s KnowledgePanel), incorporating both random-digit dialing and address-based sampling to ensure representation of households with and without landline phones and providing Internet access and hardware as necessary	868 Black/African American adults	Adapted HIV Conspiracy Beliefs scale, ecological-level socioeconomic position (interpersonal and extra-organizational level SRD)	Recent HIV testing (i.e. within the last 12 months)	A stronger belief in HIV conspiracies and engaging in higher HIV risk activities were found to be significantly linked to a greater likelihood of getting tested for HIV. This association was significantly mediated by individual-level HIV risk but not by area-level socioeconomic position
Braksmajer, 2018 [64]	To investigate how race/ethnicity influences the relationship between trust in one's primary care provider and willingness to consider PrEP	Cross-sectional survey	Mid-sized city in western New York	Participants recruited using four different approaches: distribution of materials at STI testing clinics, presence at local pride events, utilization of a local HIV care and prevention agency's website, and in-person recruitment at a syringe exchange program managed by the same HIV care and prevention agency	385 heterosexual men and MSM	Sociodemographic information including race/ethnicity and education; adapted scale for trust in one's primary care provider (interpersonal level SRD)	PrEP willingness, barriers to PrEP use	Trust in primary care provider (PCP) was significantly associated with PrEP willingness. No differences were found between racial groups in the effect of PCP trust on PrEP willingness. No significant associations were found between race/ethnicity and PrEP willingness
Brooks, 2018 [42]	To examine the association between HIV/AIDS conspiracy beliefs and intention to adopt PrEP	Cross-sectional survey	Los Angeles, CA	Participants recruited from community-based organizations serving Black MSM, community presentations, internet postings on craigslist, posting on Facebook pages of CBO’s serving Black MSM, and participant referrals	224 Black MSM	Two HIV/AIDS conspiracy belief scales: genocidal, treatment-related (interpersonal level SRD)	Intention to adopt PrEP	Black MSM who agreed with the genocidal or treatment-related conspiracy beliefs scales had a lower intention to adopt PrEP
Bunting, July 2022 [17]	To investigate the effects of a patient's race and the race of their partner on the assumptions medical students make about the patient’s sexual behavior, adherence, and HIV risk when seeking PrEP	Experimental survey study	United States	Details about the study were shared with students from 16 U.S. medical schools (10 allopathic and 6 osteopathic) with a total enrollment of 12,660 students. The initial information message intentionally did not reveal the study’s emphasis on HIV and PrEP to minimize the potential for selection bias	1472 U.S. medical students (allopathic and osteopathic)	Implicit racism measured with the Implicit Association Test (interpersonal level SRD)	Assumptions about patients seeking PrEP, willingness to prescribe PrEP	In the case of MSM patients, having a Black partner was linked to a greater assumption of patient non-adherence to PrEP compared to having a White partner. In the case of women who have sex with men, White women were assumed to have higher likelihoods of condomless and extra-relational sex, nonadherence to PrEP, and a greater HIV risk. Implicit racism did not show any connection with negative assumptions about Black patients compared to White patients based on patient or partner race. Implicit racism was only correlated with anticipated condomless sex
Bunting, Dec 2022 [18]	To investigate the interactive effect of patient race and clinician implicit racism on the indirect association between assumed patient condom use and clinician willingness to prescribe PrEP	Experimental survey study	United States	Details about the study were shared with students from 16 U.S. medical schools (10 allopathic and 6 osteopathic) with a total enrollment of 12,660 students. The initial information message intentionally did not reveal the study's emphasis on HIV and PrEP to minimize the potential for selection bias	600 medical students (allopathic and osteopathic)	Implicit racism measured with a modified version of the Implicit Association Test (interpersonal level SRD)	Assumptions about patients seeking PrEP, willingness to prescribe PrEP	Black MSM were perceived as more likely to adhere to PrEP compared to White MSM. There was no significant difference in the willingness to prescribe PrEP between Black and White MSM
Bunting, 2023 [19]	To assess the impact of PrEP/HIV knowledge, implicit racism, and heterosexism of pharmacy students on their assumptions about a patient's behavior (e.g., condomless sex, adherence, extra-relational sex) and their confidence in providing PrEP-related care	Experimental survey study	United States	Participants were recruited using a convenience sampling method. Study information was distributed to students by administrators at 4 U.S. pharmacy schools	180 pharmacy students	Implicit racism measured with the Implicit Association Test (interpersonal level SRD)	Assurance in delivering care related to PrEP for the patient in the following areas: (1) issuing a PrEP prescription to the patient, (2) advising the patient about PrEP, (3) guiding the patient on general HIV risk reduction, and (4) overseeing the patient's care during PrEP usage	Black patients were assumed to be less adherent to PrEP if prescribed compared to White patients. There were no differences in assumptions of sexual risk behaviors if prescribed PrEP and confidence providing PrEP-related care between Black and White patients. Implicit racism was associated with lower confidence providing PrEP-related care. PrEP/HIV knowledge, implicit sexual orientation bias, and assumed sexual risk behaviors if prescribed PrEP were not associated with confidence providing care
Burns, 2021 [65]	To assess whether factors at a structural level (e.g., neighborhood features), separate from individual attributes, can forecast PrEP participation among Black MSM	Cross-sectional survey	Jackson, MS and Baltimore, MD	Participants were recruited from a wide range of community settings, including HIV clinics, AIDS Service Organizations, community-based organizations, social media, word of mouth, and also using social networks of participants	142 Black MSM	Health Empowerment Scale, Emotional Support Scale, income level, education level, homelessness in the last 12 months, history of incarceration, insurance, health behavior (interpersonal and extra-organizational level SRD)	PrEP utilization (i.e. had taken PrEP within the previous 12 months)	Black MSM living in a zip code with a greater proportion of residents below the poverty line and those facing housing insecurity, and individuals with higher health empowerment scores are less inclined to consider PrEP use
Calabrese, 2014 [20]	To explore medical students’ stereotypes about sexual risk compensation among Black versus White MSM and the impact on willingness to prescribe PrEP	Cross-sectional survey	Northeastern United States	Participants recruited through an email requesting participation and containing the survey link	102 medical students	Racial bias while making clinical judgements (interpersonal level SRD)	Willingness to prescribe PrEP	Black patients were rated as more likely to engage in increased unprotected sex if prescribed PrEP compared to White patients, and medical students were less willing to prescribe PrEP to these patients
Calabrese, 2016 [36]	To assess how public attitudes towards PrEP differed based on social group	Cross-sectional survey	United States	Participants recruited through two Internet-based survey platforms	154 members of the general public	Explicit racism measured using Modern Racism Scale (interpersonal level SRD)	PrEP knowledge	Racism was associated with greater heterosexism. Racism and heterosexism were associated with lower respect for taking PrEP, lower support for PrEP financial assistance, greater predicted risk compensation, lower perceived community support for access, and lower support for PrEP financial assistance
Calabrese, 2018 [16]	To examine associations between biases (racism and heterosexism) and PrEP clinical decision-making, and to explore prior PrEP education as a potential buffer	Cross-sectional survey	Northeastern United States	Participants recruited via mass email to all students enrolled at two medical schools using internal email distribution lists	115 medical students	Explicit racism measured using Modern Racism Scale; Implicit Racial Prejudice assessed using a standard set of verbal stimuli that participants classified as ‘Good’ or 'Bad' (intra-organizational level SRD)	Willingness to prescribe PrEP	When racism was operationalized as explicit racism or implicit racial prejudice, there was no interaction effect of race and racial bias on clinical judgment. When racism was operationalized as risk-related racial stereotypes, Black patients were judged as more likely to engage in extra-relational sex than the White patient among medical students who more strongly endorsed risk-related racial stereotypes. Explicit racism was correlated with exhibiting stronger risk-related racial stereotypes, judging the patient as more likely to engage in extra-relational sex, and reported lower intention to prescribe. Implicit racial prejudice and implicit risk-related stereotypes were positively correlated with one another but unrelated to intention to prescribe PrEP
Calabrese, 2022 [14]	To examine practicing providers’ biases in PrEP clinical decision-making based on patient race, sexual orientation, and injection drug use	Cross-sectional survey	United States	Participants recruited through mass email to US providers via professional email distribution lists (American Academy of HIV Medicine, Society for General Internal Medicine)	370 licensed providers practicing in HIV or primary care settings (physician, nurse, PA); 190 participants met IAT duration and accuracy criteria	Modern Racism Scale for provider explicit racial prejudice; Race Implicit Association Test for implicit racial prejudice (interpersonal level SRD)	Willingness to prescribe PrEP	In the restricted sample, there were no significant effects of patient race on the outcomes and no moderating effects of provider explicit or implicit racial prejudice. In the larger sample, Black patients' requests for PrEP were judged as more important than those of White patients. Additionally, Black patients were judged as more responsible, and judging a Black patient as more responsible was associated with a higher intention to prescribe PrEP
Caponi, 2019 [61]	To outline the demographic features of patients identified as at high risk for HIV infection in a healthcare environment focused on underserved populations and to assess the distinctions between individuals who have received PrEP prescriptions and those who have not	Cross-sectional study	New York, NY	Data collected from EMR from a substantial urban network of federally qualified health centers (FQHC) known as the Community Healthcare Network (CHN)	9659 patients	Sociodemographic information including race, ethnicity, and poverty level (extra-organizational SRD)	PrEP prescription by primary care providers	Out of 4,248 eligible Black/African American individuals in the study, 8.7% were prescribed PrEP, in contrast to 30.6% and 43.1% among White and Asian individuals, respectively. Among the 3,828 Hispanic/Latinx participants, 21.7% received PrEP, while 17.7% of those not identifying as Hispanic/Latinx (5,831 participants) were prescribed PrEP
D’Avanzo, 2019 [68]	To evaluate how and to what extent medical mistrust presents a barrier to trans women’s awareness and knowledge of PrEP as a conditional element toward PrEP use	Cross-sectional survey	Greater Philadelphia area	Participants recruited through active and passive means, the majority through chain recruitment	78 transgender women	Sociodemographic information including race/ethnicity, education, and income; opinions about medical providers, attitudes about healthcare (interpersonal and extra-organizational level SRD)	PrEP knowledge, intention to use PrEP, past PrEP use	Greater PrEP concern and medical mistrust were observed among the cluster of mostly White trans women with greater educational attainment. The cluster of mostly Black trans women with lower educational attainment was more likely to have heard of PrEP from a doctor and be more comfortable in healthcare settings. There were no significant differences in intention to use PrEP and differences in past PrEP use between clusters
Doherty, 2022 [11]	To investigate the effect of racism and social determinants of health on PrEP-to-Need Ratio, a measure of sufficient PrEP use	Retrospective analysis	United States	Data from online datasets including AIDSVu.org, CDC HIV surveillance data, U.S. Census Bureau, and Symphony Health	696 counties	Percent African American and percent Hispanic populations in a county	The PrEP-to-Need Ratio (calculated by dividing the number of PrEP prescriptions by the count of HIV diagnoses within the county)	Percent African American and percent uninsured were negatively correlated with PrEP-to-Need Ratio (PNR). Positively correlated with PNR were median household income and severe housing cost burden. For low populations, percent African American, percent uninsured, and severe housing cost burden were significant exposures. For large populations, median household income, percent in poverty, percent uninsured, and percent African American were significant
Eaton, 2014 [37]	To assess the relationship between willingness to use PrEP and PrEP knowledge and use, health care access experiences, race-based medical mistrust, sexual partners and behaviors, and drug and alcohol use	Cross-sectional survey	Southeastern United States	Participants recruited through venue intercept procedures at a community event	398 Black HIV negative MSM	Adapted Group-based Medical Mistrust Scale (assesses previous experiences of racial discrimination as well as feelings of discomfort and suspicion toward healthcare personnel and pharmacological treatment), healthcare access experiences (interpersonal level SRD)	PrEP knowledge, PrEP use, willingness to use PrEP	Barriers to willingness to use PrEP included discomfort with talking to a health care provider about having sex with men, not having discussed having sex with men with a healthcare provider, and race-based medical mistrust. People with higher race-based medical mistrust scores were less likely to use PrEP. Not being comfortable talking about sexual health with a provider was associated with less willingness to use PrEP. Residing within city limits was associated with less willingness to use PrEP
Eaton, 2017 [49]	To assess the relationships between potential barriers to PrEP (PrEP stigma and conspiracy beliefs) and interest in PrEP	Cross-sectional survey	Southeastern United States	Participants recruited through venue intercept procedures at a large gay pride festival	85 BMTW and 179 WMTW	Sociodemographic information including race/ethnicity; adapted scales regarding PrEP stigma and PrEP conspiracy beliefs (interpersonal and extra-organizational level SRD)	PrEP knowledge, PrEP use	Conspiracy beliefs related to PrEP were more frequently reported among BMTW than WMTW. Responses to PrEP stigma belief items did not significantly differ across BMTW and WMTW. BMTW were found to have less interest in using PrEP if they were more likely to believe that PrEP was for promiscuous individuals
English, 2020 [66]	To examine associations between incarceration history, police and law enforcement discrimination, and recent arrest with sexual HIV risk, willingness to initiate PrEP, and psychological distress among Black SMM	Cross-sectional survey	United States	Participants recruited from baseline data of the Understanding New Infections through Targeted Epidemiology study (UNITE), which recruited participants through advertisements on social media (e.g., Facebook) and sexual networking sites/applications (e.g., Adam4Adam, Black Gay Chat)	1172 Black SMM	Sociodemographic information including incarceration history and recent arrest; police and law enforcement discrimination (extra-organizational level SRD)	PrEP knowledge, willingness to use PrEP	Incarceration and recent arrest were associated with greater sexual HIV risk. Incarceration, police and law enforcement discrimination, and low socioeconomic status were associated with lower PrEP willingness
Fitch, 2021 [69]	To explore, in a climate of expanded healthcare access, the demographic, behavioral, structural, and psychosocial differences between MSM who did and did not report using PrEP in the past 12 months	Cross-sectional survey	Boston, MA	Data from the National HIV Behavioral Surveillance system (venue-based and time-spaced samples)	530 MSM total	Sociodemographic information including race/ethnicity, insurance status, access to healthcare, and history of incarceration (extra-organizational level SRD)	PrEP use	History of incarceration, limited access to healthcare, lack of health insurance, and not being “out” to a healthcare provider were all associated with not using PrEP. Race/ethnicity was not significantly associated with PrEP use in the past 12 months
Galletly, 2019 [58]	To investigate whether participant self-reports of never having undergone an HIV test were linked to misunderstandings about the immigration implications tied to seeking healthcare, getting tested for HIV, and receiving an HIV diagnosis	Cross-sectional survey	Raleigh-Durham region of North Carolina	Participants recruited through informal communication and the distribution of flyers by Spanish-speaking Latino research team members to institutions that catered to Latino migrants, including churches, healthcare centers, and agencies offering support and information to migrants. Recruitment also took place at locations where English as a second language classes and naturalization preparation courses were being held	297 Latino migrants	Hispanic Stress Inventory-Immigrant form (Cervantes 1991), Acculturative Stress Scale (Arbona 2010), and items developed to assess beliefs about the immigration ramifications of being tested for HIV (extra-organizational level SRD)	Ever receiving an HIV test	Those who had never undergone HIV testing, in comparison to those who had been tested, reported a greater number of misunderstandings about immigration laws and policies. There was no significant link between the history of HIV testing and concerns about deportation. The scale measuring misunderstandings about immigration laws and policies was a robust predictor of not getting tested for HIV. On average, for every additional misconception held by a participant, the likelihood of never having undergone an HIV test nearly doubled
García, 2017 [46]	To develop an instrument that measured Latino MSM attitudes and beliefs towards PrEP, identify associations between demographic factors and PrEP related factors, and to suggest culturally appropriate strategies for the promotion of PrEP among the Latino MSM population	Cross-sectional survey	San Antonio, TX	Participants were found through an anonymous survey distributed on the Internet	159 Latino MSM, age 21–30	Sociodemographic information including household income and education (extra-organizational level SRD)	PrEP awareness, PrEP use	For Latino MSM, lower educational attainment and lower levels of reported annual household income were significantly associated with lower levels of PrEP awareness. Greater levels of education and household annual income were significantly associated with reporting current PrEP use
Grieb, 2013 [55]	To investigate the factors associated with HIV testing in the last half-year concerning housing security and frequent changes in residence (relocating two or more times in the past six months)	Cross-sectional survey	Baltimore, MD	Participants were gathered through specific street-level outreach efforts, the distribution of promotional materials, and recommendations from healthcare facilities and community organizations	620 low-income urban African Americans	Sociodemographic information including educational attainment, housing situation and stability, housing transience, and depression using the Center for Epidemiological Depression Scale (Radloff, 1977) (extra-organizational level SRD)	HIV testing within the past 6 months	Unstably housed participants had a 1.4 times higher likelihood of having received an HIV test in the past 6 months. However, when accounting for other variables, housing stability was not found to be linked to recent HIV testing. On the other hand, individuals who were currently homeless were twice as likely to have undergone recent HIV testing. After adjusting for other variables, individuals who were transient were 1.7 times more likely to have undergone HIV testing in the past 6 months. Additionally, those currently homeless were 2.5 times more likely to have been recently tested for HIV when compared to those who were not homeless, even when accounting for transience
Hamilton, 2022 [62]	To examine disparity of PrEP use within patients receiving PrEP through Planned Parenthood League of Massachusetts	Cross-sectional study	Massachusetts	Sample drawn from deidentified electronic health records from patients who had sought medical care at Planned Parenthood League of Massachusetts	1114 PrEP users	Sociodemographic information including race and ethnicity (extra-organizational SRD)	PrEP Prescription	Individuals identifying as Asian had a significantly different mean PrEP prescription (M = 4.40) compared to those identifying as Black (2.76), White (3.43), Other Race (2.97), and Patient Declined (2.51)
Harkness, 2023 [72]	To assess the factors influencing the engagement of South Florida-based LSMM in PrEP and behavioral health treatment and the significance of these factors in predicting PrEP engagement and behavioral health engagement	Cross-sectional survey	Miami, FL	Participants gathered via social media, mailing lists, community gatherings, snowball referrals, and an opt-in database for contact	290 LSMM	5-item Everyday Discrimination Scale (to assess experiences of discrimination related to various aspects of identity, including income and immigration status) (interpersonal and extra-organizational level SRD)	PrEP engagement (measured by stages of change cascades consisted of five “levels” with stages of change theory: pre-contemplation, contemplation, preparation, initiation, and maintenance)	Stigma about treatment and discrimination related to income and immigration status were found to be determinants of PrEP treatment involvement. Individual level elements included knowledge and self-efficacy. Structural determinants included financial stress
Haubrick, 2023 [78]	To explore the connections between racism and homophobia and the motivation and comfort levels of young Southern Black MSM when it comes to obtaining PrEP in different settings	Cross-sectional survey	Jackson, MS	Participants recruited from three STI/HIV testing clinics through the distribution of flyers and referrals by word-of-mouth to complete the survey	65 BMSM	Questions adapted from the Homophobia Scale (Diaz 2001, to assess experiences of homophobia and racism as an adult) (interpersonal level SRD)	PrEP motivation and self-efficacy by general level of comfort receiving PrEP at any of the seven location types	Individuals who experienced significant levels of racism expressed greater comfort with receiving PrEP through mail home-delivery when contrasted with those who reported low level of experiences of racism
Hojila, 2021 [27]	To identify gaps in the PrEP care continuum, evaluate demographic and clinical factors associated with attrition, and characterize HIV infection incidence at each step of the PrEP continuum	Retrospective cohort study	Kaiser Permanente Northern California health network	Data extracted from electronic health record	13,906 adults	Sociodemographic information including race/ethnicity, insurance type, and neighborhood deprivation index (extra-organizational level SRD)	PrEP prescription, initiation, discontinuation, and reinitiation	African American and Latinx patients were less likely to receive a PrEP prescription, less likely to initiate PrEP, and more likely to discontinue PrEP compared to White patients. Those with lower SES were less likely to be prescribed PrEP, less likely to initiate PrEP, and more likely to discontinue PrEP
Hoyt, 2012 [21]	To explore the connections between institutional mistrust (comprising systematic discrimination, organizational suspicion, and conspiracy beliefs), behaviors associated with HIV risk, and HIV testing in a diverse group of MSM, and to examine whether the perceived likelihood of contracting HIV plays a mediating role in these relationships, with a focus on both White and ethnic minority MSM	Longitudinal prospective cohort study	Central Arizona	Participants recruited through advertisements in the community and media, direct outreach, and the snowball method	399 MSM	Institutional Mistrust Scale (to assess the extent of mistrust towards institutions involved in HIV/AIDS matters, such as medical services and government) (interpersonal level SRD)	HIV testing status (ever tested vs. never tested)	In ethnic minority MSM only, greater levels of organizational suspicion was related to less likelihood of having been tested for HIV. Additionally, greater levels of systematic discrimination and conspiracy beliefs were significantly related to increased likelihood of engaging in sexual risk behavior
Huang, 2018 [63]	To highlight gaps in effective PrEP implementation efforts	Cross-sectional study	United States	Data from IQVIA database about antiretroviral drug prescriptions dispensed	32,853 people eligible for PrEP	Sociodemographic information including race/ethnicity and insurance type (extra-organizational SRD)	PrEP prescription	From 2014 to 2016, nearly six times as many White people were prescribed PrEP, compared to Black, Hispanic, and Asian people
Hull, 2021 [15]	To explore provider willingness to prescribe PrEP under the influence of racially biased clinical judgements	Experimental survey study	48 hotspot counties in the United States designated in the Ending the Epidemic initiative	Providers recruited through the Qualtrics Medical Professional Panel	160 healthcare providers with prescribing privileges, who were aware of PrEP, and who served patient populations that include adult Black women	Color-Blind Racial Attitudes Scale (3 subscales; Unawareness of Racial Privilege, Institutional Discrimination, Blatant Racial Issues) (intra-organizational level SRD)	PrEP prescription	Providers who scored high on a modern racism measure were less willing to discuss and prescribe PrEP to Black patients. These effects were mediated by provider perceptions of patients’ abilities to adhere to PrEP but were not mediated by their expectations of risk-compensatory behaviors
Irvin, 2014 [54]	To explore the link between perceived racial discrimination specifically related to healthcare and its impact on access to healthcare services (measured by healthcare utilization) and HIV testing among Black MSM who were HIV-negative	Cross-sectional survey	Atlanta, GA; Boston, MA; Los Angeles, CA; New York, NY; San Francisco, CA; and Washington, DC	Participants recruited from the community or through referrals from index participants who were part of the same sexual networks	1167 Black MSM	Healthcare-specific racial discrimination (interpersonal level SRD)	Having received an HIV test in the previous year	Compared to those not reporting healthcare-specific racial discrimination, a higher proportion of participants reporting healthcare-specific racial discrimination had at least partial college education, were unemployed, and had healthcare insurance. Perceived racial discrimination within healthcare settings was positively associated with recent healthcare visits and HIV testing in the preceding year
Kalichman, 2023 [67]	To assess the effects of intersectional stigma on PrEP outcomes using a newly developed approach that conceptualizes intersection in geometric terms	Cross-sectional survey	United States	Participants recruited through social media platforms (Facebook, Jack'd, TikTok)	422 Black sexual minority men	Enacted (24-itmes) and anticipated (17-items) stigma scales with attributions to race and sexual minority status and developed from pilot studies (interpersonal level SRD)	PrEP interest, barriers to PrEP	Racial attributions for enacted stigma and the intersection of racial-sexual minority status for enacted stigma were significantly associated with greater medical mistrust. Anticipated stigma attributed to race and the intersection of racial-sexual minority status predicted less PrEP interest and perceived barriers to using PrEP for HIV prevention
Kanny, 2019 [73]	To examine racial/ethnic disparities in MSM regarding PrEP use and awareness	Cross-sectional survey	Atlanta, GA; Baltimore, MD; Boston, MA; Chicago, IL; Dallas, TX; Denver, CO; Detroit, MI; Houston, TX; Los Angeles, CA; Memphis, TN; Miami, FL; Nassau and Suffolk counties, NY; New Orleans, LA; New York, NY; Newark, NJ; Philadelphia, PA; Portland, OR; San Diego, CA; San Francisco, CA; San Juan, PR; Seattle, WA; Virginia Beach, VA; and Washington, DC	Data from 2017 National HIV Behavioral Surveillance (NHBS)	4056 MSM	Sociodemographic information including race/ethnicity and health insurance (extra-organizational level SRD)	PrEP knowledge, PrEP discussion with health care provider, PrEP use	Black and Hispanic MSM were significantly less likely than were White MSM to have discussed PrEP with a health care provider. White MSM who discussed PrEP with their health care provider were significantly more likely than were Black MSM to use PrEP. This disparity between White and Black MSM persisted among those who had health insurance and those who had a usual source of health care. Disparities in PrEP use between White and Black MSM existed in the south and west U.S. census regions, whereas disparities between White and Hispanic MSM existed only in the south
Kim, 2019 [31]	To describe geographic variation in access to PrEP providers in New York City, and compare to geographic variation of high incidence	Cross-sectional study	New York, NY	PrEP locator database used to locate PrEP-prescribing clinics and their characteristics	154 registered PrEP providers in NYC	Neighborhood sociodemographic measures including race/ethnicity, income, and insurance coverage (extra-organizational level SRD)	PrEP provider geographic distribution	PrEP providers in New York City were effectively distributed in high HIV incidence neighborhoods. Neighborhoods with a high percentage of Black and Hispanic residents, as well as residents below the federal poverty line, were not associated with lower levels of PrEP provider density. Neighborhood insurance coverage was also not associated with PrEP provider density
Kimball, 2020 [38]	To examine how medical mistrust relates to 4 stages of the PrEP cascade among Latino SMM	Cross-sectional survey	San Diego County	Participants recruited via paid advertisements on dating websites as well as registry of participants of other studies who expressed interests in future studies	151 LSMM	Group Based Medical Mistrust Scale (interpersonal level SRD)	PrEP awareness, willingness, current use, and adherence	Medical mistrust was significantly associated with decreased odds of PrEP awareness, willingness, current use, and adherence
Lechuga, 2018 [81]	To develop, pilot test, and analyze the Immigration Law Concerns Scale, which measures the influence of perceived immigration laws on Latino migrants’ HIV testing behavior	Cross-sectional survey	Metropolitan areas in two south states in South-eastern United States	Participants recruited through referrals from a network of community-based organizations serving Latinos and Spanish-language radio ads	339 Spanish-speaking non-citizen Latino migrant adults, both documented and undocumented, having lived in the US for at least 6 months	Immigration status, Immigration Law Concerns Scale (extra-organizational level SRD)	HIV testing behavior	Three distinct factors emerged where perceived immigration law was associated with HIV testing behaviors: “Concerns about the Exchange of Patient Medical Information,” “Concerns related to Public Charge Designation,” and “Concern that HIV is a Disfavored Disease.”
Lee, 2019 [59]	To investigate variations in HIV risk behaviors and HIV testing behaviors among undocumented and documented Latino immigrants, and to explore disparities in key barriers to HIV testing, including factors like socioeconomic status, access to healthcare, and apprehension about HIV testing, within these two groups	Cross-sectional survey	West Queens district of New York City	Specially trained personnel visited homes within the designated area and approached potential participants following a structured recruitment script	301 Latino immigrants	Sociodemographic information including country of origin, highest level of education, annual household income, legal status (extra-organizational level SRD)	HIV testing ever, time since their last HIV test in the last 12 months, intentions to get tested for HIV in the next 12 months, intentions to test annually	Documented and undocumented Latino immigrants had distinct sociodemographic disparities, but their current and future HIV testing behaviors were similar. Although undocumented immigrants had lower rates of ever being tested for HIV compared to documented immigrants, there were no significant differences in recent testing, intentions for testing in the next 12 months, or intentions for testing annually
Lelutiu- Weinberger, 2016 [48]	To examine differences in perceived barriers and facilitators to PrEP access for Black and Latino MSM compared to other MSM	Cross-sectional survey	New York, NY	Participants recruited through passive recruitment (i.e., display of study flyers and cards in local venues catering to our target populations; placement of study ads on websites and mobile applications used by MSM to seek partners), active recruitment (i.e., outreach at bars, events, community-based organizations), and participant referral	491 MSM	Sociodemographic information including race/ethnicity, education, income (extra-organizational level SRD)	PrEP access and uptake	Compared with other MSM, BLMSM were more likely to have public insurance and access health care via public clinics. They also regarded having to talk to their doctor about their sex life as a barrier to PrEP. Furthermore, they believed that PrEP did not provide complete protection and reported that taking a pill every day was a barrier. Additionally, they were less likely to endorse agency in medical decision making. They were more likely to consider access to free sexual health and additional supportive services to be facilitators of PrEP use. BLMSM were more likely to agree that 1-on-1 counseling for PrEP use would be a facilitator. Lastly, they were more likely to use text-based support and group adherence as significant facilitators of PrEP use
Li, 2019 [50]	To examine the variation in PrEP awareness, PrEP use, and willingness to use PrEP between counties in terms of county- and individual-level factors	Cross-sectional survey	United States	Data from sample of MSM collected in the American Men’s Internet Survey	8338 MSM aged 15 and older	Sociodemographic information including race/ethnicity, education, and current health insurance; neighborhood-level factors including percent Black male, median household income, percent uninsured, and GINI coefficient measuring regional income inequality (extra-organizational level SRD)	Awareness of, willingness to use, and use of PrEP	At the county level, median household income and income inequality were associated with PrEP awareness, but percent of population who were Black male was not associated. At the individual level, PrEP awareness was higher among those who were White (versus Black), had a college degree, had current health insurance, or had higher yearly income. Differences in willingness to use PrEP could not be attributed to county-level variables. Increased odds of using PrEP was associated with increased median household income
Liu, 2022 [53]	To assess the socioecological determinants of HIV prevention services uptake (e.g., HIV testing, PrEP) and HIV-related risk behaviors (e.g., sexual practice, substance use) among young MSM	Cross-sectional survey	Nashville, TN and Buffalo, NY	Participants recruited through community outreach infrastructures of HIV service organizations (flyer distribution, peer referral, local LGBT event interception, social media posts, venue outreach, recruitment during regular HIV clinic visits)	209 YBMSM and 109 YWMSM	Sociodemographic information including race, education, food security, housing stability, and health insurance (extra-organizational level SRD)	Awareness of, willingness to use, and history of use of PrEP	Among YBMSM, structural inequities (housing instability, food insecurity, internalized homonegativity) were positively associated with anxiety, depression, and stress. Anxiety and depression were associated with increased alcohol/drug use before sex, while stress was associated with reduced recent HIV testing and PrEP awareness, willingness, and use. Among YWMSM, psychological buffers (perceived social support, resilience) were associated with reduced anxiety, depression, and stress. Anxiety was associated with increased condomless insertive/receptive anal sex and recent HIV testing
Lozano, 2023 [74]	To identify determinants of LSMM PrEP use and HIV testing and examine differences across subgroups (age and immigration history)	Cross-sectional survey	Greater Miami, FL area	Participants recruited through snowball recruitment, community venues, social media, listservs, and "consent-to-contact" database	256 adult LSMM	Sociodemographic information including nativity and immigration history; two newly developed scales to assess barriers and facilitators to PrEP and HIV testing (intraorganizational and extra-organizational level SRD)	PrEP knowledge and use, HIV testing	PrEP barriers with the highest endorsement were lack of knowledge about PrEP, mistrust and concerns, costs and insurance issues, privacy concerns, and lack of perceived need or urgency for PrEP; LSMM under 40 rated lack of perceived need or urgency for PrEP significantly higher than older LSMM. There was a statistically significant difference in language and immigration barriers to PrEP between the immigration history subgroups. PrEP facilitators with the highest endorsement were PrEP affordability, perceived benefits, knowledge, navigation support, and providers having a positive demeanor; LSMM under 40 rated PrEP affordability and PrEP being normalized by others significantly higher compared to older LSMM. There were no differences across subgroups of immigration history in terms of facilitator endorsement
Maksut, 2018 [44]	To determine the extent to which Black MSM were aware of PrEP, whether participants’ age moderated the relationship between perceived healthcare-related discrimination and PrEP awareness, and whether age moderated the relationship between disclosure of same-sex behavior to a healthcare provider and PrEP awareness	Cross-sectional survey	Southeastern United States	Participants were recruited from gay-identified bars, clubs, bathhouses, parks, and street locations, from online classifieds, and on social media	147 Black MSM	Adapted perceived healthcare-related discrimination questionnaire (Wilson and Yoshikawa, 2007) (interpersonal level SRD)	PrEP awareness	BMSM who had not heard of PrEP scored higher on the perceived healthcare-related discrimination measure and were less likely to have talked to healthcare providers about having sex with men. When age was a mediator, perceived healthcare-related discrimination was significantly and negatively associated with PrEP awareness, and the relationship strengthen as age increased beginning at 30.2 years of age
Mauldin, 2022 [32]	To describe PrEP referral and HIV/STI prevention networks among organizations that serve MSM	Cross-sectional survey	Houston, TX and Chicago, IL	Sampling strategy not reported; data from a prospective cohort study (YMAP: Young Men's Affiliation Project)	68 organizations involved in HIV/STI prevention efforts including PrEP-prescribing healthcare providers, community-based organizations (CBOs), and social establishments and organizations	Survey assessing organization characteristics (including racial composition), ties, and networks (intra-organizational level SRD)	PrEP provider distribution and connection	The majority of organizations conducted PrEP awareness/promotion activities, but fewer made PrEP referrals, with little overlap between the collaboration and referral networks. Networks tended to have a densely connected core group of organizations with associated peripheral organizations, and the peripheral organizations had relatively few ties among/between themselves. In Houston's collaboration network, higher Black-serving organizations tended not to hold as influential positions for controlling communications or flows of resources
Mustanski, 2018 [22]	To explore the role that PrEP stigma, and its associated individual and geospatial factors, may play in the lives and decisions about PrEP use among young MSM and young transgender women	Longitudinal prospective cohort study	Chicago metropolitan area	Participants recruited from previous cohorts of sexual and gender minority youth and YMSM (as part of RADAR study), through being a serious romantic partner OR peer of an existing cohort member	620 Young MSM and trans women, aged 16–20	Sociodemographic information including race/ethnicity and gender identity (extra-organizational level SRD)	PrEP knowledge, awareness, and stigma	Stigma scores were higher in Black and Latino participants (compared to White participants) as well as participants identifying as male (compared to participants not identifying as male). Participants with prior knowledge about PrEP reported lower stigma and higher positive attitudes. Also, PrEP stigma had significant geospatial clustering: hotspots were identified in neighborhoods with high HIV incidence and concentration of racial minorities, while coldspots were identified in areas with high HIV incidence and low LGBT stigma
Ojikutu, 2014 [56]	To understand barriers to HIV testing for non-US born Black individuals	Cross-sectional survey	Massachusetts counties of Suffolk, Essex, Plymouth, and Middlesex	Participants recruited through convenience sampling through 41 recruitment events held at national celebration events, health fairs, ethnic grocery stores, shopping centers, night clubs/bars, community health centers, and churches, in conjunction with a community-based mobile health van	555 adult immigrants from sub-Saharan Africa and the Caribbean	Sociodemographic information including immigration status, education, and housing; (extra-organizational level SRD)	Knowledge and testing history of HIV; barriers-to-HIV-testing scale (Awad 2004)	Primary language other than English, lower education, low income, no regular provider, and recent immigration were independently associated with greater barriers to HIV testing. Barriers due to health care access, privacy, fatalism, and anticipated stigma were greater for recent versus longer-term immigrants
Ojikutu, 2019 [33]	To determine the association between spatial access to clinics where PrEP is prescribed and willingness to use PrEP	Cross-sectional analysis	United States	PrEP clinics and patients found through ZIP code analysis and individual-level data obtained from 2016 National Survey on HIV in the Black Community	787 Black adult individuals and 760 distinct ZIP codes	Sociodemographic information including education and insurance; locations and density of clinics where PrEP is prescribed (from AIDSVu.org) (extra-organizational level SRD)	Willingness to use PrEP, PrEP provider distribution	26% of participants were willing to use PrEP. 38% of participants had to drive more than 1 h to access a PrEP provider. Participants living in areas with higher PrEP clinic density were significantly more willing to use PrEP: 1 SD higher density of PrEP clinics per 10,000 people was associated with 16% higher willingness. Participants with a high school diploma or GED were less likely to be willing to use PrEP than participants without such education levels. Self-reported high HIV risk and residence in the West compared to Northeast were significantly associated with higher likelihoods of willingness to use PrEP
Ojikutu, 2020 [75]	To determine the association between PrEP willingness and HIV-related medical mistrust	Cross-sectional survey	United States	National Survey on HIV in the Black Community administered online between February and April 2016, participants drawn from an online web panel	522 Black women, 347 with expanded PrEP indications	Sociodemographic information including ethnicity, income, and education; HIV-related mistrust and belief in HIV conspiracy theories (interpersonal and extra-organizational level SRD)	Knowledge of and willingness to use PrEP	Women with high HIV conspiracy belief scores were more willing to use PrEP. Among scale items, “there is a cure for HIV but the government is withholding it from the poor” and “HIV is a man-made virus” were associated with willingness to take PrEP. In the bivariate analysis, lower income, less well educated, and unmarried/non-cohabiting women were more willing to take PrEP
Okafor, 2017 [23]	To understand the socio-structural and behavioral correlates of PrEP use among a sample of high-risk HIV-negative MSM in Los Angeles	Longitudinal prospective cohort study	Los Angeles, CA	Participants were recruited from an ongoing study (MSM & Substance Use cohorts at UCLA Linking Infections, Noting Effects (mSTUDY))	185 MSM	Sociodemographic information including insurance and housing status (extra-organizational level SRD)	PrEP use	Having health insurance coverage was associated with recent PrEP use. Unstable housing was associated with lower PrEP use
Olansky, 2020 [40]	To examine HIV conspiracy beliefs and PrEP awareness among Black and Hispanic/Latino MSM	Cross-sectional survey	Chicago, IL; Fort Lauderdale, FL; and Kansas City, MO	Participants recruited through online, agency, and street outreach	438 Black MSM and 439 Hispanic/Latino MSM age 18 or older	HIV/AIDS conspiracy belief scale (interpersonal level SRD)	PrEP awareness	Men reporting HIV/AIDS conspiracy beliefs were less likely to be aware of PrEP. HIV-negative men were less likely to report awareness of PrEP if they held a HIV/AIDS conspiracy belief. Among HIV-positive men, HIV/AIDS conspiracy beliefs were inversely associated with awareness of PrEP
Pitasi, 2021 [28]	To assess trends in new HIV infections, prevention, and treatment outcomes among MSM in the United States, compared by race and age	Retrospective cohort study	California, Delaware, Florida, Georgia, Illinois, Indiana, Michigan, Mississippi, New Jersey, New York, North Carolina, Oregon, Pennsylvania, Puerto Rico, Texas, Virginia, and Washington	Data from National HIV Surveillance System (NHSS), National HIV Behavioral Surveillance (NHBS), and Medical Monitoring Project (MMP, which recruited participants in person, by telephone, or by mail	4466 MSM	Sociodemographic information including race/ethnicity and age (extra-organizational level SRD)	PrEP knowledge and use	Among MSM with a likely PrEP indication, Latino MSM and Black MSM were less likely to have discussed PrEP with a health care provider and less likely to have used PrEP within the past 12 months compared to White MSM
Pyra, 2022 [26]	To explore patterns of PrEP use over the first year after initiation, and patient characteristics and experiences at baseline and over follow-up, specifically among racial/ethnic minority patients of all genders	Retrospective cohort study	Howard Brown Health Center, Chicago	Data from electronic health records of patients who started PrEP from 2015–2018	2159 patients who started PrEP in 2015–2018 and self-identify as Latinx, Asian, or Black	Sociodemographic information including race/ethnicity and insurance (extra-organizational level SRD)	PrEP adherence	Black patients were more likely to be on the short use trajectory or declining use trajectory. Residents of Westside (majority Latinx population) were more likely to be on the short use trajectory or declining use trajectory. Residents of Southside (majority Black population) were more likely to be on the short use trajectory. People with public health insurance were more likely to be on the short use trajectory than the sustained use trajectory
Quinn, 2023 [77]	To examine the relationship between intersectional discrimination and PrEP use among YBSMM and TW, and the role of resilience, social support, and Black LGBTQ community connectedness as potential mediators	Cross-sectional survey	Milwaukee, WI and Cleveland, OH	Participants recruited through in-person avenues at community organizations and social venues frequented by YBSMM, social media postings, and paid advertising on the dating/hookup smartphone apps Jack’d and Grindr	283 adult YBSMM and TW	Intersectional Discrimination Index (Scheim and Bauer, 2019), measured experiences of interpersonal homophobia and racism (Jeffries et al., 2013), LGBT People of Color Microaggressions scale (newly developed items to assess PrEP social concerns) (interpersonal level SRD)	Current and future PrEP use	Individuals with higher levels of anticipated discrimination were less likely to be current PrEP users. Higher levels of daily discrimination were associated with an increased likelihood of using PrEP in the future and higher levels of resilience, social support, and connection to the Black LGBTQ community. Social support mediated the effect of day-to-day discrimination on likelihood of future PrEP use. There was a significant and negative indirect effect of PrEP social concerns on current PrEP use via Black LGBTQ community connectedness
Ramírez-Ortiz, 2021 [60]	To determine the prevalence of self-report HIV testing pre- and post-immigration and the associations between pre-immigration HIV sexual risk behaviors, access to healthcare post-immigration, and HIV testing post-immigration among young adult recent Latino immigrants	Cross-sectional analysis	Miami-Dade County, FL	Participants recruited using respondent-driven sampling, seeds were recruited via flyers and in-person throughout neighborhoods and businesses with substantial recent Latino immigrant populations, community-based agencies serving recent Latino immigrants, and during Latino health fairs, and each seed (recruiter) was asked to refer 3 individuals from their social network who met eligibility criteria	540 young adult Latino immigrants (age 18–34) who immigrated within 12 months prior to baseline with the intention of staying at least 3 years after baseline	Sociodemographic information including documentation status and time living in the United States (extra-organizational level SRD)	HIV testing pre- and post-immigration	23.8% of participants reported HIV testing post-immigration and 56.7% reported HIV testing pre-immigration; proportions varied by country/region of birth. The main reasons for not getting tested for HIV post-immigration were low perception of HIV risk and not having been offered an HIV test. The prevalence ratio for post-immigration HIV testing was higher for participants who had health insurance and/or a regular doctor or healthcare provider after immigration. Post-immigration HIV testing was higher for participants who had ever been tested for HIV before immigration. The prevalence ratio was lower for those who engaged in condomless sex in the three months prior to immigration
Saleska, 2021 [76]	To explore racial/ethnic differences in PrEP use by geographic setting among adolescent cisgender MSM	Cross sectional study; data from randomized control trial within Adolescent Medicine Trials Network (NIH-funded clinical trial)	New Orleans, LA and Los Angeles, CA	Participants recruited from community-based organizations and clinics serving gay, bisexual, and transgender youth, homeless youth, and post-incarcerated youth, as well as on social media and through referrals	729 adolescents who identified as cisgender MSM	Sociodemographic information including race/ethnicity and employment (extra-organizational level SRD)	PrEP use	The odds of PrEP use among Black adolescents were considerably lower than White adolescents in New Orleans, although no there was no evidence of differences in Los Angeles. In unadjusted analysis, the odds of PrEP use among Black adolescents was less than one quarter that of White adolescents
Sarno, 2022 [24]	To investigate the conflict between sexual orientation and racial/ethnic identities as a mechanism linking minority stress to HIV-related outcomes among MSM and transgender women and gender nonbinary (TGN) POC	Longitudinal prospective cohort study	Chicago, IL	Participants recruited through in-person and online methods including participants from previous cohorts and serious partners/peers of enrolled participants	337 sexual and gender minority assigned male at birth (SGM-AMAB) individuals, ages 16–29	Conflicts in Allegiances Scale (measure of conflict between one’s racial/ethnic identity and sexual orientation identity) (interpersonal level SRD)	PrEP use	The associations between identity conflict at Time 2 and PrEP use at Time 3 and between internalized heterosexism (IH) at Time 1 and PrEP Use at Time 3 were not significant. There was not a significant indirect effect from IH to PrEP use through identity conflict. There were no significant associations between identity conflict at Time 2 and PrEP use at Time 3 or concealment at Time 1 and PrEP use at Time 3. The indirect effect was not significant. Microaggressions were not significantly associated with PrEP use, and there was no significant indirect effect from microaggressions to PrEP use through identity conflict
Schumacher, 2021 [29]	To evaluate PrEP delivery overall and relative to community need	Retrospective cohort study	Baltimore, MD	Data obtained using public health surveillance from 7 clinical sites participating in a health department-led demonstration project to increase PrEP	25,866 PrEP-screened individuals	Sociodemographic information including race/ethnicity (extra-organizational level SRD)	PrEP referrals, PrEP prescription	Non-Hispanic Black patients were less likely to be linked or referred to PrEP care and less likely to be prescribed PrEP compared to Non-Hispanic White patients
Shrader, 2023 [34]	To examine associations between LSMM’s immigration status and Spanish-language PrEP service availability	Cross-sectional survey	South Florida	Participants recruited through respondent-driven sampling while PrEP service navigators were identified using the CDC PrEP Directory	131 LSMM	Zip code data, location data for Spanish-speaking PrEP navigator services from CDC National Prevention and Information Network (extra-organizational level SRD)	PrEP provider distribution	Latin American-born LSMM had 0.09 times lower availability of Spanish language PrEP navigation services within 1 mile relative to their US-born counterparts. Zip-code-level HIV risk was associated with 1.7 times higher odds of Spanish PrEP service availability within 1 mile. Spanish-language PrEP navigation services tended to be located in high HIV incidence zip codes where LSMM reside, signifying a matched need, particularly along Miami-Dade’s coastal municipalities
Siegler, 2018 [35]	To explore distribution of publicly listed PrEP-providing clinics in the US	Cross-sectional study	United States	PrEP locator database used to locate PrEP-prescribing clinics and their characteristics	2094 publicly listed, PrEP-providing clinics in the United States	County-level and regional sociodemographic information including proportion of residents uninsured or living in poverty (extra-organizational level SRD)	PrEP provider distribution	Counties with higher proportions of their residents living in poverty, lacking health insurance, identifying as African American, or identifying as Hispanic/Latino saw differences between disease burden and service provision. The Southern region of the US accounts for over half of all new HIV diagnoses but only one-quarter of PrEP-providing clinics
Taggart, 2020 [51]	To examine awareness and willingness to use PrEP among sexually active black and Latinx adolescents	Cross-sectional survey	New York, NY; Washington, DC; Newark NJ; Philadelphia PA; and Baltimore MD	Participants from NY and DC recruited through community efforts (flyers in youth servicing organizations and community health clinics, direct recruitment in adolescent primary care clinics and emergency departments, and pediatric physician referral) while participants from NJ, PA, and MD recruited through a Qualtrics panel distributed to adolescents residing in those areas	208 Black and Latinx adolescents (13–17 years old)	Sociodemographic information including race/ethnicity and education (extra-organizational level SRD)	PrEP awareness and willingness	38% of participants reported PrEP awareness, which was associated with Black race and prior HIV testing. PrEP willingness was reported by 22% of the sample and was associated with higher age, more education, having had condomless sex in the past 6 months, perceived likelihood of acquiring HIV, and PrEP awareness
Tekeste, 2019 [39]	To investigate the role of medical mistrust in the relationship between women’s race and comfort discussing PrEP with a healthcare provider	Cross-sectional survey	3 cities in Connecticut	Participants recruited through Planned Parenthood reproductive health centers	501 HIV negative adult women who had no experience with PrEP and were heterosexually active	Sociodemographic information including race/ethnicity, education, and employment; Group-Based Medical Mistrust Scale (interpersonal and extra-organizational level SRD)	Interest in learning more about PrEP, intentions to use PrEP	Black women reported higher medical mistrust compared to White women. Black women also reported more interest in learning more about PrEP and greater intention to use PrEP if it were available for free
Turpin, 2019 [57]	To identify latent classes of HIV testing probabilities based on combinations of healthcare access, diagnosed depression, and poverty, and to determine if latent classes of HIV testing deviate from unidimensionality	Cross-sectional survey	United States	Participants recruited through telephone survey	1,786 heterosexual Black men	Sociodemographic information including education, poverty status, and health insurance (extra-organizational level SRD)	HIV testing	Participants were categorized into 4 classes according to various syndemic factors: Class 1 was characterized by low proportions of all risk factors; Class 2, relatively high healthcare barriers and most likely to have no routine checkup in the past year but had relatively low depression diagnosis and poverty; Class 3, moderately high depression diagnosis and the highest proportion of poverty, but generally low barriers to healthcare access including the lowest proportions of not having a routine checkup in the past year; Class 4, high proportions of all the risk factors, with the highest proportions of depression diagnosis, poverty, not having health insurance, not having a personal doctor, and not having been able to access a doctor due to cost. Class 4 was least likely to have been HIV tested (38.0%) followed by class 3 (56.6%), class 2 (64.7%) and class 1 (70.0%)
Turpin, 2021 [25]	To test if both racism and homophobia were associated with HIV testing in a U. S. sample of BSMM and BTW with a history of incarceration	Longitudinal prospective cohort study	Atlanta, GA; Boston, MA; Los Angeles, CA; New York City, NY; San Francisco, CA; and Washington, DC	Participants recruited through direct field-based outreach, engagement of key informants and community groups, advertising through various print and online media, and the use of chat room outreach and social networking sites	655 Black, African American, Caribbean Black, or multiethnic Black people who self-identify as man or being male at birth	Racism and Life Experiences Scales (Harrell, 1997); one question asking about number of times the participant spent one or more nights in jail or prison; reported race (interpersonal level SRD)	HIV testing	Both racism and homophobia were associated with lower HIV testing, though their interaction was associated with unexpectedly higher HIV testing
Walsh, 2023 [52]	To examine the amount and type (sources and perceived tone) of information individuals receive about PrEP and its potential influence on PrEP uptake	Cross-sectional survey	Milwaukee, WI	Participants recruited by research staff at a table in a heavily trafficked	331 sexual and gender minorities who have sex with men (SGMSM)	Sociodemographic information including race/ethnicity and material hardship (extra-organizational level SRD)	PrEP stigma, intentions, discussion with a health care provider, and use	Black participants were more likely to have heard about PrEP from healthcare providers, their families, newspapers, magazines, TV shows, movies, or music compared to White participants. Black participants had heard about PrEP from more sources overall (4.51 sources on average) than White participants (3.75). Black participants were more likely to describe the tone of messages they received about PrEP (including from social media) as negative or neutral (vs. positive) than White participants. This was true across all sources considered and for the overall perceived tone. Black participants and participants with indications for PrEP use were more likely to have discussed PrEP with a healthcare provider and to be PrEP users
Watson, 2022 [71]	To assess the demographic impact of PrEP-related stigma and logistical barriers on current PrEP use among Black and Hispanic/Latinx SMMTW	Cross-sectional analysis	United States	Participants recruited from national networks, listservs, social media (Twitter, Facebook, and Instagram), local community-based organizations, health departments, and other health centers to advertise the survey	827 Black and Hispanic/Latinx SMMTW, aged 18–29	Sociodemographic information including race/ethnicity, education level, and income (extra-organizational level SRD)	PrEP-related internalized stigma, PrEP barriers, PrEP use	Participants with higher levels of education reported lower PrEP-related internalized stigma, lower logistical barriers, and higher rates of current PrEP use. Participants who worked full time reported lower PrEP-related internalized stigma, lower logistical barriers, and higher rates of current PrEP use
Whitfield, 2020 [70]	To examine the relationship between condom and PrEP use and internalized racism	Cross-sectional survey	Los Angeles, CA; San Francisco, CA; Denver, CO; Ft. Lauderdale, FL; Chicago, IL; New Orleans, LA; Boston, MA; Baltimore, MD; Charlotte, NC; New York, NY; Memphis, TN; Nashville, TN; Austin, TX; Dallas, TX; Houston, TX; Atlanta, GA; and Washington, DC	Participants recruited face-to-face in Denver, Chicago, New York, Dallas, Atlanta, and Washington, DC, while web-based recruitment on facebook and Grindr was used to recruit participants in Atlanta, Austin, Houston, Dallas, new Orleans, Los Angeles, Chicago, Baltimore, Boston, Nashville, Memphis, San Francisco, Ft. Lauderdale, Charlotte, and Washington, DC	436 BMSM	Nadanolitization Scale to examine internalized racism (Goffman, 2009; Pescosolido et al., 2008) (interpersonal level SRD)	PrEP knowledge and use	Internalized racism was not significantly associated with condom use among Black MSM engaging in insertive anal sex or receptive anal sex. Higher levels of internalized racism were associated with higher levels of PrEP use, although not statistically significant. Men who reported full-time employment or did not report drug use were more likely to report current PrEP use
Xavier Hall, 2022 [30]	To assess PrEP-related outcomes (eligibility, first prescription, second prescription) across race and sexual orientation of cisgender Latino men	Retrospective cohort study	Chicago, IL	Data extracted from electronic health record	8271 men who identified as cisgender and Latino and were HIV-negative at the time of the first recorded visit	Sociodemographic information including race and monthly income; clinic location (extra-organizational level SRD)	PrEP prescription	Latino-only participants had lower odds of PrEP eligibility and PrEP 1st prescription compared to White Latino participants. Participants seeking care at North Chicago clinics had lower odds of PrEP 1st prescriptions, compared to other clinics. Participants making less than $823/month were less likely to be PrEP eligible and had lower odds of PrEP 1st prescription compared to those making $824-$3343/month
Zhou, 2021 [45]	To explore the moderating effects of race, ethnicity and homelessness on the relationship between provider trust and PrEP use	Cross-sectional study	United States	Participants recruited using clinic-based advertisements and flyers, word-of-mouth, and direct referral from their counselors	234 opioid-dependent patients on methadone who met eligibility criteria for PrEP	Sociodemographic information including race/ethnicity and homelessness; Provider trust (interpersonal and extra-organizational level SRD)	PrEP use	Mean provider trust was significantly higher among Black vs White patients. Though race/ethnicity was not a significant moderator on provider trust and PrEP use, increased provider trust was marginally associated with increased PrEP use among Black patients. Homelessness significantly moderated trust and PrEP use, and provider trust among non-homeless participants was positively correlated with PrEP use

MSM: Men who have sex with men; BLMSM: Black and Latino men who have sex with men; BMTW: Black men and transgender women who have sex with men; WMTW: White men and transgender women who have sex with men; SMM: sexual minority men; GBMMS: Group Based Medical Mistrust Scale; SMMTW: Sexual minority men and transgender women; LSMM: Latino sexual minority men

### Overview of Results

Most studies (50) employed cross-sectional analyses to investigate the impact of SRD on the PrEP care continuum, employing questionnaires and self-reported measures to gather data on participants’ experiences and perspectives. Four experiments evaluated participant reactions and biases towards different patient situations [15, 17–19]. Five studies utilized a longitudinal prospective cohort methodology [21–25], and six adopted a retrospective cohort approach [11, 26–30]. Lastly, six studies examined the geographic distribution of PrEP clinics, which allowed for a direct assessment of SRD at the extra-organizational level [11, 31–35].

### Measurements of SRD

Studies exhibited significant heterogeneity in their measurement of SRD. A total of 17 studies used validated measures to assess SRD exposures. The Modern Racism scale was used in three studies, which is a measure of covert racial prejudice and attitudes [14, 16, 36]. The Group-Based Medical Mistrust Scale measures health care-related trust with a specific focus on the context of racism and discrimination [37–39]. Two studies utilized the HIV/AIDS conspiracy belief scale [40, 41], while another study created two scales adapted from the original scale to measure “genocidal” and “treatment-related” conspiracy beliefs [42]. Four studies used the Racial Implicit Association Test [14, 17–19], while another study used three subscales from the Color-Blind Racial Attitudes Scale [43]. Ten studies created and validated new scales or adapted questions from previous studies to create new unvalidated tools. Most studies captured sociodemographic information about participants, and some studies relied solely on self-reported race as the exposure of interest, without further measurement of specific forms of SRD.

PrEP outcome measures, recorded in Table 1, were heterogeneous but not completely mutually exclusive across studies and are listed in detail below.

### PrEP Knowledge and Awareness

Heightened medical mistrust was associated with reduced comfort in discussing PrEP with healthcare providers and a lower odds of PrEP awareness [38, 39]. Likewise, perceived healthcare-related discrimination was negatively linked to PrEP awareness and to a reduced likelihood of talking to providers about having sex with men [44]. The inverse was true as well: increased provider trust was associated with increased PrEP awareness and use among Black patients. For Latino MSM specifically, lower educational attainment and lower levels of reported household income were associated with decreased PrEP awareness [45]. For Black and Latino men and Black TW, incorrect beliefs about PrEP, concerns about side effects, and belief in conspiracy theories negatively correlated with PrEP awareness and knowledge [40, 42, 46–48]. Some studies found that race/ethnicity, residential instability, and incarceration were not associated with decreased PrEP awareness but that increased awareness was positively associated with higher education, access to insurance, identifying as Black, and prior HIV testing [46, 49–51]. Similarly, for young Black MSM compared to young White MSM, food insecurity was associated with increased levels of stress, which was associated with reduced PrEP awareness [52]. In summary, SRD at the interpersonal level (medical mistrust, perceived discrimination) and extra-organizational level (low education, low health literacy, low income, poor access to insurance and food) were negatively associated with PrEP knowledge and awareness [53].

### HIV Testing

The association between SRD exposures at the three levels and HIV testing were mixed: some studies showed that belief in HIV conspiracies and perceived racial discrimination were associated with higher HIV testing [41, 54], while other studies showed that patient experiences of racism and homophobia, perceived systematic discrimination in access to or policies regarding HIV-related services were associated with decreased HIV testing. [21, 25] At the extra-organizational level, some studies showed that homelessness or anxiety associated with food insecurity and housing instability were associated with higher HIV testing [52, 55] while other studies showed that primary language other than English, lower education, lower income, lack of a regular healthcare provider, and lack of insurance were associated with decreased HIV testing [56, 57]. For Latinx immigrants, misunderstanding about immigration laws and policies, being undocumented, recent immigration, having a low perception of risk, and not being offered an HIV test post-immigration were associated with decreased or no HIV testing [56, 58–60].

### PrEP Prescription and Initiation

People self-identifying as White or Asian had higher rates of PrEP prescription [61–63], while those self-identifying as African American or Latinx or having lower income had lower rates of PrEP prescription [27, 29, 30]. Willingness to use PrEP was positively associated with a variety of sociodemographic factors and SRD exposures, including higher age, higher educational level, trust in primary care provider, PrEP awareness, perceived likelihood of acquiring HIV, and living in an area with higher PrEP clinic density [33, 50, 64]. Decreased willingness to initiate PrEP was associated with extra-organizational SRD, such as low socioeconomic status, living in neighborhood with greater proportion of residents below poverty line, low education, residing within city limits, housing instability, and history of incarceration [33, 37, 52, 65, 66]. At the interpersonal and extra-organizational level, experiences of discrimination by police and law enforcement, anticipated racial stigma, and identities at the intersection of racial-sexual minority status were associated with decreased willingness to initiate PrEP [66, 67]. Negative exposures specific to the healthcare setting included higher race-based medical mistrust scores, medical mistrust in general, discomfort talking about sexual health with a provider, and having conspiracy beliefs [37, 38, 42, 68]. Intra-organizational SRD was demonstrated by the fact that healthcare providers and medical students who scored high on modern racism measures were less inclined to prescribe PrEP to Black patients [15, 16]. Black patients were sometimes assumed to be non-adherent [17, 19] and other times judged as more responsible [14, 18], which had negative and positive effects on the intention to prescribe, respectively. Medical students were less willing to prescribe PrEP to Black MSM compared to White MSM due to concerns about sexual risk compensation, where a decrease in the perceived risk of getting HIV (due to taking PrEP) may lead to increased HIV risk behaviors [20].

### PrEP Use

Facilitators for PrEP use included greater levels of education, full-time employment, greater annual household income, and having health insurance [23, 35, 45, 49, 69–72]. Black patients who heard about PrEP from a variety of external sources (e.g. family, friends, healthcare providers, media) were more likely to use PrEP [51]. On the other hand, White patients who discussed PrEP with a provider were more likely to use PrEP than Black patients who discussed PrEP with a provider [73]. For Latino SMM specifically, facilitators were PrEP knowledge, PrEP affordability, previous HIV testing, healthcare navigation support, and positive provider demeanor [39, 47, 60, 71, 74], encompassing exposures at all three levels of SRD.

Regarding decreased PrEP use, extra-organizational level SRD, such as housing instability, homelessness, history of incarceration, limited access to healthcare, and lack of insurance, were associated with decreased PrEP uptake [23, 46, 52, 53, 69, 72]. Individuals experiencing unstable housing conditions were less likely to utilize PrEP effectively [30, 75]. Low socioeconomic status was associated with lower rates of PrEP use even after being prescribed [28, 29, 45]. When examining race/ethnicity, Black and Latino MSM, compared to White MSM, had lower levels of PrEP use, and PrEP use among Black adolescents differed geographically, with 6% of Black adolescents using PrEP in New Orleans and 11% in Los Angeles [28, 76]. Lower access to PrEP clinics was associated with decreased PrEP use in under-resourced neighborhoods, which were communities with predominantly Black and Latinx residents as well as populations below the federal poverty line [35]. At the interpersonal level, income and immigration status discrimination and higher levels of anticipated discrimination were associated with less PrEP use [24, 72, 77]. Notably, experienced racism was associated with greater comfort in receiving PrEP through mail-home-delivery [78]. Barriers to use were anticipated racial stigma, having to take a pill every day, having to talk to their doctor about their sex life, medical mistrust, cost concerns, insurance issues, and lack of knowledge [38, 39, 47, 48, 67, 74]. Latinx immigrants faced further barriers, including experienced discrimination related to immigration status and privacy concerns [56, 72, 74].

### PrEP Adherence and Retention

PrEP adherence decreased as levels of medical mistrust experienced by patients increased [38, 73]. Additionally, PrEP adherence varied between different neighborhoods with neighborhoods predominantly composed of Black and Latinx communities exhibiting lower rates of adherence [26]. Moreover, two studies demonstrated that higher rates of PrEP discontinuation were associated with lower socioeconomic status and utilization of public health insurance [26, 27]. Surprisingly, one study found that higher levels of daily discrimination were associated with an increased likelihood of future PrEP use as well as higher levels of resilience and social support [77]. Similar to the outcome of PrEP knowledge and awareness, this stage of the continuum was associated with SRD at the first level (medical mistrust, discrimination) and third level (neighborhood composition, socioeconomic status, public health insurance usage).

## Discussion

These studies provide valuable insights into the complex dynamics and systemic factors influencing access, utilization, and outcomes within the PrEP care continuum. The diverse array of study designs utilized contributed to a comprehensive understanding of the impact of SRD on the PrEP care continuum, encompassing both individual experiences and broader structural factors. The use of validated measures provided a robust foundation for examining the impact of SRD on PrEP outcomes and allowed for more nuanced analyses. On the other hand, self-reported surveys provided direct insight into participants’ experiences. In some studies, self-reported race/ethnicity was not associated with PrEP care continuum outcomes but instead was associated with SRD at multiple levels (i.e., provider trust, history of incarceration, limited access to healthcare, lack of health insurance, the stigma associated with disclosing sexual orientation to a healthcare provider). The mixed results regarding the association between race/ethnicity and PrEP care continuum outcomes demonstrate the importance of understanding how contextual factors, rather than race/ethnicity itself, affect PrEP health services utilization and outcomes.

We found that medical mistrust was the factor most associated with a lack of PrEP knowledge and awareness. Medical mistrust was often measured alongside and correlated with measures of perceived discrimination and belief in conspiracy theories; the suggested relationship between these three exposures raises the need to understand how these constructs (in isolated and aggregate form) affect the patients’ experiences with the healthcare system. Additionally, discussing PrEP with a healthcare provider seem to differ for patients within the same race due to factors including age, geography, and gender identity [28, 51, 68, 73]. Although standards for clinical research and patient protection have significantly improved in past decades, more work needs to be done to address the negative impact of medical mistrust on patient care. Our findings emphasize the need to address medical mistrust as a form of SRD that hinders several steps along the PrEP care continuum, especially PrEP knowledge and awareness.

Of the papers that examined PrEP prescription and initiation, the prominent SRD exposure was racial bias in both medical trainees and PrEP providers. At this point in the continuum, patients have had contact with the healthcare system and have received HIV testing and their providers are aware that they may benefit from PrEP. Studies show that the concept of “risk compensation” was often the reason that providers would not prescribe PrEP to their patients. This reasoning was mostly applied to racial/ethnic minority patients and usually not to their White counterparts and persists despite ample evidence that PrEP use does not result in behavior change or an increase in sexually transmitted infections [79]. This bias is a form of SRD that is deeply rooted in the medical system, as evidenced by its presence at different levels of medical training. The incorrect assumptions about risk perception and behavior in racial minorities hinder PrEP prescription and initiation. Further, the lack of proactive prescribing and engagement with racial/ethnic minority populations leads to limited opportunities for PrEP discussions and education, further hindering awareness levels. Together, these findings suggest an imperative need for more informed medical decision-making to improve PrEP prescription and initiation. Putting it all together, negative perceptions of Black patients influencing clinical decisions exemplify SRD at the interpersonal level. Racial differences in the likelihood of discussing PrEP with a healthcare provider can be classified as both first-level SRD as well as second-level SRD, as this may be due to a lack of organizational procedures and protocols in place to ensure that patients in need are not deprived of PrEP.

Social determinants of health have a notable association with PrEP uptake and use. The association between lower socioeconomic status (SES) and lower PrEP uptake can be attributed to the fact that Black and Latinx individuals are more likely to belong to lower SES groups. Similarly, Black and Latinx communities have been and continue to be disproportionately affected by housing instability due to the historical legacy of slavery and racial discrimination, including housing discrimination, in the U.S. Another important social determinant is the inequitable distribution of PrEP-provider clinics, which disproportionately affects Black and Latinx communities. Because PrEP has shown efficacy in decreasing HIV incidence in regions with the greatest PrEP uptake [80], this discrepancy suggests the need for further examination of the complex interplay between clinic accessibility as SRD and PrEP uptake in under-resourced communities. Addressing these forms of SRD is crucial for promoting equitable PrEP access and utilization, reducing health disparities, and achieving health equity for all populations.

We found that PrEP adherence and retention were negatively impacted by medical mistrust and social determinants of health, both of which also influenced earlier stages of the PrEP care continuum. The fact that medical mistrust has an impact this far down the continuum demonstrates that medical mistrust works at different levels of the healthcare system and highlights the importance of building trust and effective patient-provider relationships. Additionally, the presence of socio-economic disparities and unequal access to healthcare resources contribute to challenges faced by marginalized communities, ultimately affecting their ability to adhere to PrEP regimens effectively. This highlights the significant role that neighborhood factors and SES play in PrEP adherence and retention. These findings emphasize the need for targeted interventions addressing both interpersonal and extra-organizational racism to enhance PrEP adherence and ensure equitable access and utilization of this preventive measure.

One population highlighted by our study was that of Latinx immigrants, who had unique barriers to PrEP use, including experienced discrimination regarding immigration status and privacy concerns [56, 72, 74]. Since Spanish language PrEP navigation services are often less geographically accessible for Latin American-born Latino sexual minority men compared to their US-born counterparts [34], it is important to point out the finding that navigation support is a facilitator to PrEP use [74]. Combining PrEP education with existing educational efforts focused on healthcare access and legal protections as an immigrant may be even more useful, as misunderstandings about immigration laws and policies were associated with participants having never undergone HIV testing [58, 81].

## Limitations

Although we followed a systematic method, it is possible that not all the relevant articles were captured and incorporated into our review. Additional potential limitations are excluding studies published in languages other than English, excluding purely qualitative studies (done to examine the population-level impact of SRD), and restricting the search to articles available in the PubMed and PsycINFO databases. Furthermore, the possibility of publication bias and the inherent limitations of the studies’ methodologies may impact the generalizability of the findings. Lastly, the scope of our paper did not include the effects of gender discrimination, though the studies were reviewed included a diversity of groups within the Lesbian, Gay, Bisexual, Transgender, and Queer community. It is important to note this limitation, as gender discrimination can intersect with racial discrimination and play a role in PrEP outcomes. However, to maximize the pool of included studies, we also searched the references of included studies for additional eligible articles.

## Public Health Implications

Overall, the results of this systematic review highlight the profound impact of SRD on the PrEP care continuum. SRD, at multiple levels, acts as a significant barrier to PrEP knowledge and awareness, prescription and initiation, uptake and use, and adherence and retention among racial and ethnic minority populations. These findings underscore the urgent need for targeted interventions, policy changes, and comprehensive approaches that address SRD and its detrimental effects on PrEP access and utilization among marginalized communities. By addressing SRD, healthcare systems can promote equitable PrEP care and contribute to the overall goal of reducing HIV transmission and achieving health equity for all populations.

## Supplementary Information

Below is the link to the electronic supplementary material.

Supplementary file1 (DOCX 16 KB)
