# Supplementary material for: Assessing Structural Racism and Discrimination Along the Pre-exposure Prophylaxis Continuum: A Systematic Review
Source: AIDS Behav. 2024 Jun 8;28(9):3001–37. doi: 10.1007/s10461-024-04387-y (PMC11390845; doi:10.1007/s10461-024-04387-y)
Supplement: Supplementary file 1 — Supplementary file1 (DOCX 16 KB) [file 10461_2024_4387_MOESM1_ESM.docx]

**Supplemental Material: Search Strategies**

We first designed a search for PubMed and later adapted it for PsychINFO, looking for publications from 2012 with terms related to HIV, PrEP, and structural racism. For our systematic review, we followed a structured process, carefully screening and combining relevant literature using Covidence, ensuring a thorough and unbiased analysis based on predefined criteria.

**PubMed:**

(HIV OR HIV Infections OR Acquired Immunodeficiency Syndrome OR HIV/AIDS OR HIV Infections OR HIV OR Human Immunodeficiency Virus OR AIDS Virus OR Acquired Immune Deficiency Syndrome Virus OR Acquired Immunodeficiency Syndrome Virus OR AIDS OR Acquired Immunodeficiency Syndrome OR Acquired Immunodeficiency Syndrome OR People living with HIV OR Persons living with HIV OR PLWHA OR PLHIV)

AND

(Pre-Exposure Prophylaxis OR pre-exposure prophylaxis OR preexposure prophylaxis OR PrEP OR HIV PrEP OR HIV pre-exposure prophylaxis OR HIV preexposure prophylaxis OR pre-exposure antiretroviral prophylaxis OR pre exposure antiretroviral prophylaxis OR pre-exposure chemoprophylaxis OR preexposure chemoprophylaxis OR anti-HIV prophylaxis OR pre-exposure prophylaxis OR preexposure prophylaxis OR pre exposure prophylaxis OR tenofovir OR Truvada OR emtricitabine OR descovy)

AND

(Race Factors OR Ethnic Groups OR Transients and Migrants OR Refugees OR Race OR Races OR Ethnicity OR Ethnic Group OR American OR Americans OR Black OR Brown OR African American OR Afro American OR Asian American OR Asian Americans OR Asian Americans OR Latino OR Latinos OR Hispanic OR Hispanics OR Latina OR Latinas OR Latinx OR Spanish OR Mexican OR Indian OR Indigenous OR First Nation OR Native OR Alaskan OR Alaskans OR Pacific Islander OR Transients OR Migrants OR Immigrants OR Emigrants OR Alien OR Foreigner OR Refugee OR Asylum OR Displaced OR Minorities OR People of color OR Minority Health)

AND

(Race Relations OR Prejudice OR Social Discrimination OR Xenophobia OR Healthcare Disparities OR Aggression OR Stereotyping OR Social Stigma OR Social Marginalization OR Racism OR Race Relations OR Prejudice OR Discrimination OR Racial OR Racialized OR Implicit Bias OR Explicit Bias OR Racial-ethnic bias OR Racial-ethnic biases OR Unconscious bias OR Xenophobia OR Microaggressions OR Unfair treatment OR Oppression OR Healthcare Disparity OR Healthcare Disparities OR Health Care Inequality OR Health Care Inequalities OR Racial disparities OR Ethnic disparities OR Health disparities OR Discriminatory attitudes OR Race equity OR Health Equity OR Critical race theory OR Maltreatment OR Aggression OR Stereotyping OR Stigma OR Inequalities OR Equity OR Social Marginalization OR Historical medical assaults OR Medical mistrust OR Racialized medical services OR Discriminatory medical services OR Cultural Competency)

**PsychINFO:**

MAINSUBJECT.EXACT("AIDS (Attitudes Toward)") OR MAINSUBJECT.EXACT("AIDS Prevention") OR MAINSUBJECT.EXACT("AIDS")

AND

MAINSUBJECT.EXACT("Pre-Exposure Prophylaxis") OR MAINSUBJECT.EXACT("HIV Testing")

AND

MAINSUBJECT.EXACT("Race and Ethnic Discrimination") OR MAINSUBJECT.EXACT("Critical Race Theory") OR MAINSUBJECT.EXACT("Systemic Racism") OR MAINSUBJECT.EXACT("Racism") OR MAINSUBJECT.EXACT("Internalized Racism") OR MAINSUBJECT.EXACT("Implicit Bias") OR MAINSUBJECT.EXACT("Racial Bias") OR MAINSUBJECT.EXACT("People of Color") OR MAINSUBJECT.EXACT("Discrimination") OR MAINSUBJECT.EXACT("Social Discrimination") OR MAINSUBJECT.EXACT("Race and Ethnic Discrimination") OR MAINSUBJECT.EXACT("Minority Groups") OR MAINSUBJECT.EXACT("Minority Stress") OR MAINSUBJECT.EXACT("Prejudice") OR MAINSUBJECT.EXACT("Equity") OR MAINSUBJECT.EXACT("Health Disparities") OR MAINSUBJECT.EXACT("Racial and Ethnic Relations") OR MAINSUBJECT.EXACT("Blacks") OR MAINSUBJECT.EXACT("Asians") OR MAINSUBJECT.EXACT("Latinos/Latinas") OR MAINSUBJECT.EXACT("Alaska Natives") OR MAINSUBJECT.EXACT("Hawaii Natives") OR MAINSUBJECT.EXACT("Indigenous Populations") OR MAINSUBJECT.EXACT("Pacific Islanders") OR MAINSUBJECT.EXACT("American Indians") OR MAINSUBJECT.EXACT("Pacific Islanders") OR MAINSUBJECT.EXACT("Immigration") OR MAINSUBJECT.EXACT("Undocumented Immigration") OR MAINSUBJECT.EXACT("Refugees") OR MAINSUBJECT.EXACT("Stigma") OR MAINSUBJECT.EXACT("Microaggression")
